# Core muscle strength and stability-oriented breathing training reduces inter-recti distance in postpartum women

**DOI:** 10.1515/med-2025-1164

**Published:** 2025-02-28

**Authors:** Chao Chen, Huai-ming Zhang, Lei Shen, Hua-ping He, Zhen-yu Ma, Yu-qin Zhu, Zhao-hui Geng, Yan-xia Qin

**Affiliations:** Department of Rehabilitative Medicine, Shanghai Pudong New Area Tang Zhen Community Health Service Center, Shanghai, 201210, China; Department of Prevention and Control, Shanghai Yangpu District Mental Health Center, Shanghai, 200093, China; College of Nursing, Shanghai University of Traditional Chinese Medicine, Shanghai, 201203, China; Department of Rehabilitative Medicine, Shanghai Pudong New Area Tang Zhen Community Health Service Center, No.75 of Innovation Middle Road, Pudong New Area, Shanghai, 201210, China

**Keywords:** breathing training, core muscle strength, diastasis recti abdominis, inter-recti distance, postpartum women

## Abstract

**Objective:**

The aim of this study was to examine the impact of stability-focused core muscle strength training coupled with respiratory techniques on diminishing the inter-recti distance (IRD) among postpartum women.

**Methods:**

A total of 106 women diagnosed with diastasis recti abdominis within 42 days following delivery were 1:1 randomly allocated into a control group (*n* = 53) and an intervention group (*n* = 53). Both groups underwent standard postpartum follow-up care and independently pursued self-directed rehabilitation exercises. Additionally, the intervention group commenced a 6-week program of core muscle strength stability-oriented breathing training 42 days postpartum. Measurements of IRD were taken both pre- and post-intervention.

**Results:**

Prior to the intervention, no statistically significant difference in IRD was observed between the two groups (*P* > 0.05). Subsequent to the intervention, notable reductions in IRD measurements were evident above, at the level of, and below the umbilicus in both groups compared to baseline measurements (intervention group: *P* < 0.001 at all measured points; control group: *P* = 0.035 above the umbilicus, *P* < 0.001 at the level of, and below the umbilicus). However, the intervention group exhibited a more pronounced decrease in IRD at all measured points when compared to the control group, demonstrating statistical significance with *P* = 0.000.

**Conclusion:**

Core muscle strength stability-oriented breathing training demonstrates efficacy in reducing IRD among postpartum women.

## 1 Introduction

Diastasis recti abdominis (DRA) is characterized by the parting of the rectus abdominis muscles along the linea alba, with the gap exceeding 2 cm [1]. Patients diagnosed with DRA often experience abdominal laxity and protrusion following its onset. This condition not only compromises aesthetic appearance but also undermines spinal stability, precipitating lower back discomfort, and impeding trunk rotation capabilities [2]. Furthermore, it exerts additional pressure on the pelvic floor, potentially disrupting the physiological functions of pelvic floor organs leading to pelvic organ prolapse, thereby impacting the normal functioning of the abdominal wall [3,4]. In severe instances, DRA may cause abdominal herniation [5]. Left untreated or without appropriate exercise, DRA may manifest in approximately 50–60% of cases 6 weeks postpartum and in 39–45% of cases after 6 months [6]. Spontaneous recovery of DRA is rare, and noticeable improvement typically requires therapeutic intervention. Failure to promptly and effectively address DRA may lead to enduring functional impairment stemming from the separation of the rectus abdominis muscles [7]. Therefore, timely intervention following childbirth is imperative not only for enhancing the physical appearance of postpartum women but also for preventing secondary diseases.

According to the latest guideline, physiotherapy is the firsthand treatment for DRA [8]. However, there is currently very low-quality scientific evidence to recommend specific training programs in the treatment of DRA postpartum [9]. Respiratory rehabilitation training (RRT) is mainly used for the rehabilitation of respiratory system diseases. In recent years, the application of RRT in postpartum rehabilitation and other fields has been increasing. Among them, core muscle strength and stability-oriented breathing training may solve the problem of DRA from a mechanistic perspective by combining respiratory training with aerobic training, muscle strength, endurance training, etc. First, the internal core muscle group of the human abdomen includes the diaphragm above, pelvic floor muscles below, multifidus muscle behind, and transverse abdominis muscle on the anterior side. These muscles work together to maintain intra-abdominal pressure and trunk stability [10]. The diaphragm is the main respiratory muscle in the human body, but due to the compression of the fetus, the diaphragm in pregnant women cannot fully exert its function, leading to compensation by the auxiliary respiratory muscle, which in turn causes the function of the diaphragm to degrade to a certain extent, and the stability of the abdominal core muscle group decreases. Therefore, to restore the stability of abdominal core muscle strength, the first step is to strengthen the diaphragm. Second, to maintain the stability of the sagittal posture during pregnancy, the lumbar lordosis angle of pregnant women will adaptively increase, and the abdomen will present a “scissor opening” posture, causing the transverse abdominal muscle to be excessively stretched for a long time, resulting in a decrease in muscle strength [11,12].

In this study, we applied core stabilization exercises to the participation. Because the ribs, linea alba, and thoracolumbar fascia can all be stabilized by bilateral activation of the transversus abdominis. We aimed to assess the clinical efficacy of core muscle strength stability-oriented breathing training and explore whether it can have a positive effect on enhancing IRD among postpartum women.

## 2 Study participants and methods

### 2.1 General information

Postpartum women diagnosed with DRA 42 days subsequent to delivery were identified and selected at the Shanghai Pudong New Area Tangzhen Community Health Service Center from June 2021 to October 2022.

*Diagnostic criteria*: The diagnosis was made using the manual measurement method with an error margin of 0.1–0.5 cm, where patients were positioned in a supine position with knees bent at 90° and the soles of their feet flat on the ground [13]. During measurement, an abdominal curl exercise posture was maintained by the patients. Measurements were taken at the linea alba at the level of the umbilicus, 2 cm above the umbilicus, and 2 cm below the umbilicus. DRA was diagnosed if an IRD greater than 2 cm was observed at one or more of these locations.

*Inclusion criteria*: (1) A postpartum period ranging from 42 to 49 days; (2) age falling within the range of 20–40 years; (3) presence of DRA exceeding 2 cm; (4) pelvic floor muscle strength at level 3 or higher; (5) absence of prior treatment or exercise guidance received before this intervention; and (6) agree to participate in this study after explanation.

*Exclusion criteria*: (1) Patients with severe somatic diseases; (2) those experiencing significant impairment in organ function; (3) patients with psychiatric or cognitive impairments; and (4) patients diagnosed with malignant tumors.

*Dropout criteria*: (1) Individuals with changes in health status during the study period; (2) adverse reactions occurred during the study period; and (3) those who withdrew midway.

*Sample size*: According to the design of a differential test for two parallel controlled clinical trials, the clinical efficacy of postpartum female rectus abdominis muscle separation treatment was used as the evaluation endpoint. Based on literature review, *ρ*_1_ = 0.75, *ρ*_2_ = 0.90, *δ* = 0.15, *α* = 0.05, and *β* = 0.10 were taken, then bilateral *Z*_1−*α*/2_ = 1.96, *Z*_1−*β*_ = 1.282. The calculated sample size is 46 cases, and based on a 15% dropout rate, the required sample size is 53 cases. The total required sample size for the two groups is 106 cases. The sample size formula is as follows:

\[\frac{{({Z}_{1-\alpha /2}+{Z}_{1-\beta })}^{2}}{{\delta }^{2}}{[}{\rho }_{1}(1-{\rho }_{1})+{\rho }_{2}(1-{\rho }_{2})].]\]

This study used a single-blind method, with blinding for data collection, summarization, and statistical personnel but not for participants and intervention teams. A random number table was generated using SPSS 21.0 statistical software and the study participants were randomly divided into two groups. The control group (*n* = 53) underwent routine postpartum follow-up and engaged in self-directed rehabilitation training, which consisted of the following: (1) kneeling abdominal training: the body was supported with straight arms and legs, forming a 90-degree angle with the ground and between the shoulders and hips. Participants were instructed to maintain a straight back and waist, inhale while relaxing the abdomen, and exhale while forcefully contracting the abdomen inward and upward. This exercise comprised 10–15 repetitions per set, with 2 sets performed daily. (2) Standing abdominal training: participants stood upright with their backs against a wall, approximately 30 cm away. During inhalation, the lumbar spine was pressed against the wall, and the abdominal muscles were drawn in as much as possible. Upon exhalation, the body returned to its original position. This exercise involved 10–15 repetitions per set, with 2 sets completed daily. Along with the regimen followed by the control group, the intervention group (*n* = 53) commenced core muscle strength stability-oriented breathing training 42 days postpartum.

### 2.2 Intervention method

The training regimen extended over a continuous period of 6 weeks (Table 1). Training sessions were conducted three times weekly, specifically scheduled for Mondays, Wednesdays, and Fridays. Training started 1 h following meals, ensuring avoidance of a full stomach and prevention of sessions on an empty stomach. A progressive approach was used throughout the treatment process. Upon mastering control of the core muscle group with static limb positions, the training intensity was gradually escalated. Depending on the individual condition of the patient, an appropriate intensity level was selected for resistance band breathing training to enhance core muscle strength. Subsequently, progression involved integrating the transverse breathing method during dynamic limb movements and facilitating stabilization of the core muscle group under dynamic conditions.

**Table 1:** Core muscle strength and stability-oriented breathing training regimen

Week	Exercise	Intensity	Description
1	Static breathing training Transverse breathing Diaphragmatic breathing	Maximum heart rate 50–60%; 8 repetitions, 3 sets, 2-min rest between sets	Inhale through nose for 3 s, exhale through mouth for 6 s, and make a “ha” sound when exhaling (increasing exhalation resistance and reducing residual air in the lungs). Transverse breathing (core tightening inward): standing, sitting, or lying down, with hands placed beside the ribs on both sides of the chest. When inhaling, do not bulge the abdomen outward and keep shoulders down and relaxed. When exhaling, the ribs relax and converge, while the pelvic floor and abdomen contract slightly inward. Diaphragmatic breathing (strengthening the diaphragm): Lie down and place hands on the abdomen. When inhaling, keep the chest stable and feel the abdomen slowly rising upwards. Imagine the air slowly entering the body. When exhaling, the abdomen slowly sinks. Try not to move the chest and keep shoulders and neck relaxed.
	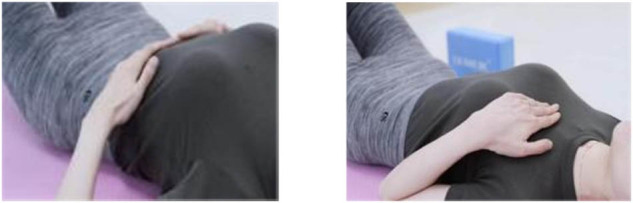		
2	Static breathing training Back breathing Pump breathing	Maximum heart rate 50–60%; 8 repetitions, 3 sets, 2-min rest between sets	Inhale through nose for 3 s, exhale through mouth for 6 s, and make a “ha” sound when exhaling (increasing exhalation resistance and reducing residual air in the lungs). Back breathing (expanding chest volume to assist breathing): prone or sitting (if breathing is difficult to control, prone position can be chosen), with palms of both hands overlapping each other, resting the forehead on the back of the hand, and relaxing the shoulders and neck. When inhaling, try to feel the opening of the back as much as possible and imagine the back becoming wider. When exhaling, slowly lower the back ribs to restore. Pump breathing (stimulating the contraction of rectus abdominis and pelvic floor muscles): sitting, naturally relax hands and place them on legs. When inhaling, keep the chest stable and feel the abdomen slowly rising upwards. When exhaling, use nose to quickly and briefly exhale, while simultaneously tightening the lower abdomen at the same frequency. Try not to move the chest and keep shoulders and neck relaxed.
	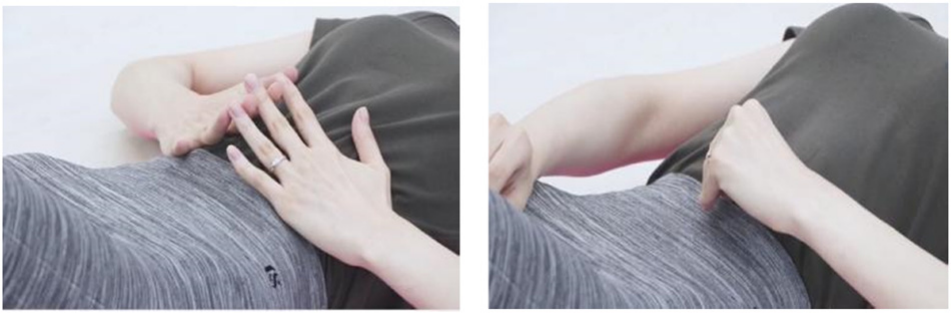		
3–4	Resistance band breathing training Strengthening transverse breathing Transversus abdominis breathing	Maximum heart rate 55–65%; 8 repetitions, 3 sets, 2-min rest between sets	Inhale through nose for 3 s, exhale through mouth for 6 s, hold still for 3 s (maintain maximum force contraction for 3 s), and make a “ha” sound when exhaling (increase exhalation resistance and reduce residual air in the lungs). Strengthening transverse breathing (strengthening core muscle strength): standing, sitting, or lying down, wrap the elastic band around the chest, and keep both arms close to the body. When inhaling, the chest expands, the ribs open laterally to both sides, the abdomen does not bulge outward, and the shoulders remain lowered and relaxed; when exhaling, the ribs relax and converge, while the pelvic floor and abdomen contract slightly inward. Transverse abdominis breathing (strengthening transverse abdominis): lie down, wrap the elastic band around the abdomen, and keep both arms close to the body. The therapist guides the patient to exhale and inhale slowly, maintaining equal inhalation and exhalation times. The abdomen is raised during inhalation and lowered during exhalation, and the elastic band is tightened during inhalation to apply resistance.
	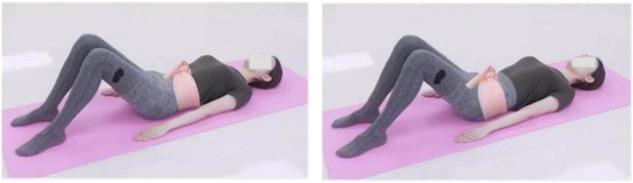		
5–6	Dynamic breathing training Pelvic clock exercise Hundreds prep exercise	Maximum heart rate 60–70%; 8 repetitions, 3 sets, 2-min rest between sets	Inhale through nose for 3 s, exhale through mouth for 6 s, and make a “ha” sound when exhaling (increasing exhalation resistance and reducing residual air in the lungs). Pelvic clock exercise (core stability control): lie in a supine neutral position, knees bent, hands placed on both sides of the body. (1) Inhale and prepare for neutral position, exhale and tilt the pelvis backward, and inhale and tilt the pelvis forwards. (2) Inhale and rotate the pelvis to the right, exhale and return to neutral position, inhale, and rotate the pelvis to the left. Hundreds prep exercise (core stability control): lie in a supine neutral position, knees bent, hands placed on both sides of the body. (1) Inhale with the right leg bent 90°, the thigh perpendicular to the ground, the calf parallel to the ground, the toes straight, and exhale while holding. (2) When inhaling again, lift the left leg and exhale while holding. (3) Prepare for inhalation, relax the shoulders, lower the ribs and pelvic floor muscles, straighten the right leg, and exhale while holding. (4) When inhaling again, return the left leg to its original position and exhale while holding. lie in a supine neutral position, knees bent, hands placed behind the neck. (1) Inhale and prepare for neutral position. Exhale and roll up the head, neck, and shoulders section by section. Inhale and straighten both hands. (2) Exhale and roll up a little higher, inhale with hands back to the neck, and return to a supine neutral position.
	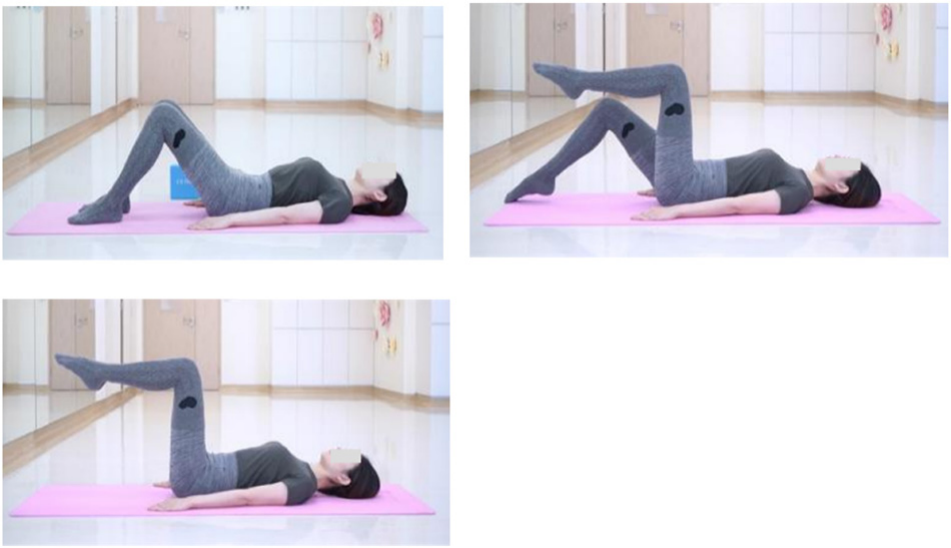		

During rehabilitation training, we will provide personalized adjustments to the intensity, difficulty, and duration time based on the physical condition of each parturient under professional guidance, while also paying attention to ongoing evaluation and feedback. Regularly evaluate the IRD and pelvic floor muscle function and monitor progress through ultrasound or electromyography. Adjust the training plan based on the evaluation results to ensure that each mother can maximize the recovery effect under safe conditions.

Specific treatment methods and arrangements are as follows:

**Static breathing training**

(1) Implementation of the transverse breathing method (inward core tightening)

Patients were positioned supine with their hands resting at their sides. During the inhalation phase, attention was focused on chest expansion, accompanied by the concurrent relaxation of the shoulders and abdomen. Conversely, during exhalation, the ribcage was permitted to relax and revert to its initial position, thereby narrowing, in tandem with the activation of the pelvic floor and the implementation of a gentle inward contraction of the abdomen. Each exercise set consisted of eight repetitions, with a total of three sets executed and a 2-min rest interval between sets.

(2) Diaphragmatic breathing (diaphragm strengthening)

Patients adopted a supine position with their hands placed at their sides. During inhalation, emphasis was placed on stabilizing the chest as the upper abdomen rose; during exhalation, gradual collapse of the upper abdomen was encouraged. Minimal movement of the chest was advocated, with relaxation of the shoulders and neck. Each exercise set consisted of eight repetitions, with a total of three sets executed and a 2-min rest interval between sets.

(3) Back breathing (thoracic volume expansion for enhanced respiration assistance)

Patients were positioned prone, their foreheads resting on the dorsa of both hands, ensuring their shoulders and neck remained relaxed. During inhalation, they were instructed to expand the back, visualizing a lateral spreading; upon exhaling, the back was to return gradually to its original state. Each exercise set consisted of eight repetitions, with a total of three sets executed and a 2-min rest interval between sets.

(4) Pumping breath (facilitating contraction of rectus abdominis and pelvic floor muscles)

Patients assumed a seated position, with their hands resting on their legs, and were instructed to maintain relaxation in the neck, shoulders, and arms. Inhalation involved focusing on chest stabilization while the abdomen gently expanded; during exhalation, a swift and forceful breath was expelled through the nose, accompanied by a rapid contraction of the lower abdomen. Each exercise set consisted of eight repetitions, with a total of three sets executed and a 2-min rest interval between sets.

**Resistance band breathing training**

(1) The strengthening transverse breathing method (core muscle strength enhancement)

In standing, sitting, or supine positions, patients utilized a resistance band encircling the chest, keeping their arms close to their torso. Inhalation involved chest expansion, along with the relaxation of the abdomen and shoulders; upon exhalation, the ribcage was allowed to relax, reverting to its original state and narrowing, while simultaneously activating the pelvic floor and performing a gentle inward contraction of the abdomen. Each exercise set consisted of eight repetitions, with a total of three sets executed and a 2-min rest interval between sets.

(2) Transversus abdominis breathing (transversus abdominis muscle strengthening)

Patients adopted a prone position, employing a resistance band wrapped around the abdominal area, with arms maintained close to the body. During inhalation, tension was applied to the resistance band, encouraging the abdomen to rise; throughout exhalation, the abdomen was allowed to lower, with the duration of inhalation and exhalation being kept equal. Each exercise set consisted of eight repetitions, with a total of three sets executed and a 2-min rest interval between sets.

**Dynamic breathing training**

(1) Advanced breathing training: pelvic clock exercise (core stability control)

While in a supine position, with knees bent and hands resting at their sides, patients employed the transverse breathing technique throughout the exercise. The procedure involved two key movements: (1) inhalation prompted a forward tilt of the pelvis, while exhalation involved tilting it backward. (2) Inhalation entailed rotating the pelvis to the right, followed by a return to a neutral position upon exhalation, and then a rotation to the left with the next inhalation. Each exercise set consisted of eight repetitions, with a total of three sets executed and a 2-min rest interval between sets.

(2) Advanced breathing training: hundreds prep exercise (core stability control)

Ⅰ: Patients adopted a supine neutral posture with knees bent and hands placed at their sides, employing the transverse breathing technique. The exercise involved several steps: (1) during inhalation, the right hip and knee were flexed to a 90° angle, positioning the thigh perpendicular to the floor and the lower leg parallel to the floor, with toes pointed, and this position was maintained during exhalation. (2) Then, on the next inhalation, the left leg was elevated and held during the subsequent exhalation. (3) The following inhalation was used to prepare for the next movement; during this phase, the shoulders remained relaxed, the ribs were lowered, and the abdominal and pelvic floor muscles were activated. This was followed by returning the right leg to its initial position and holding it there during exhalation. (4) Upon inhaling again, the left leg was returned to the starting position and maintained during exhalation.

Ⅱ: Patients adopted a supine neutral stance with knees bent and hands placed behind the neck, engaging in the transverse breathing technique. The procedure encompassed several steps: (1) upon inhaling, patients prepared their bodies in a neutral position; while exhaling, they sequentially curled up the head, neck, and shoulders, followed by an inhalation during which the arms were extended straight. (2) With exhalation, a further curl upwards was executed; during inhalation, hands were repositioned behind the neck, and the body was returned to the initial supine neutral position. Each exercise set consisted of eight repetitions, with a total of three sets executed and a two-minute rest interval between sets.

### 2.3 Observation indicators

#### 2.3.1 IRD

Patients were positioned supine with knees flexed to a 90° angle and feet flat on the surface, facilitating abdominal exposure. Their arms were crossed and placed on their shoulders. To assess, the examiner utilized the index and middle fingers to palpate perpendicularly along the abdomen’s midline. Upon the patient’s exhalation and sit-up initiation, the rectus abdominis muscles contracted, allowing the examiner to detect muscle tension bilaterally. The separation between the rectus abdominis muscles was measured at three specific points: 2 cm above the umbilicus, at the umbilical level, and 2 cm below the umbilicus.

#### 2.3.2 Pelvic floor muscle strength

Manual assessment method for pelvic floor muscle strength: The examiner positioned themselves on the right side of the patient, who was instructed to assume a supine position with knees bent and hands resting at their sides. Subsequently, the examiner inserted their index and ring fingers into the patient’s vagina and directed the patient to contract her vaginal muscles in response to verbal cues. The grading of muscle strength was based on the duration of the contraction and the number of consecutive contractions performed, as follows: Grade 0: no contraction of the vaginal muscles is discerned during examination. Grade 1: a quivering sensation in the vaginal muscles is detected. Grade 2: partial contraction of the vaginal muscles is felt, lasting 2 s, and is repeatable two times. Grade 3: full contraction of the vaginal muscles is perceived, lasting 3 s, and is repeatable 3 times, without resistance. Grade 4: full contraction of the vaginal muscles is sensed, lasting 4 s, and is repeatable four times, with slight resistance. Grade 5: Full contraction of the vaginal muscles is detected, lasting 5 s, and is repeatable five times, with sustained resistance.

### 2.4 Statistical analysis

The data underwent analysis using SPSS software version 26.0. Quantitative data are presented as mean ± standard deviation ( \[\overline{x}]\] ± SD) and analyzed using the *t*-test to compare between groups, while categorical data were expressed as percentages (%) and compared using the chi-squared (*χ* ^2^) test. A *P*-value of <0.05 was deemed statistically significant. To determine the magnitude of change, within-group and between-group effect sizes were computed for each outcome. The within-group effect size was calculated as *d* = (post mean – baseline mean)/(baseline standard deviation). The between-group effect size was calculated as *d* = ([post mean – baseline mean for the intervention group] – [post mean – baseline mean for the control group])/(pooled baseline standard deviation). Using Cohen’s effect sizes, *d* > 0.2 was considered a small effect, *d* > 0.5 a medium effect, and *d* > 0.8 a large effect.


**Informed consent:** All participants were briefed on the research, and their signed consent was obtained.
**Ethical approval:** This study was conducted in accordance with the Declaration of Helsinki. This study was conducted with approval from the Ethics Committee of Shanghai Pudong New Area Tang Zhen Community Health Service Center (2020-01).

## 3 Results

### 3.1 Demographic characteristics

A total of 106 postpartum women were included in this study and randomly divided into an intervention group and a control group. The two groups are comparable at baseline in terms of age, mode of delivery, parity, and newborn weight (Table 2). No falls occurred during the study.

**Table 2:** Demographic characteristics of participants

	Intervention group (*n* = 53)	Control group (*n* = 53)	Statistical value	*P*-value
Age (years), mean ± SD	31.86 ± 2.68	31.89 ± 2.54	0.080	0.936
Delivery mode				
Natural labor, *n* (%)	30 (56.60)	32 (60.38)	1.555	0.212
Cesarean section, *n* (%)	23 (43.40)	21 (39.62)		
Parity				
Primipara, *n* (%)	34 (64.15)	33 (62.26)	0.653	0.419
Multipara, *n* (%)	19 (35.85)	20 (37.74)		
Newborn weight (kg), mean ± SD	4.06 ± 0.48	4.11 ± 0.54	0.689	0.492

### 3.2 Comparison of pelvic floor muscle strength and waist–hip ratio recovery between the two groups

When comparing the intervention group to the control group, no statistically significant differences were observed in the changes in pelvic floor muscle strength and waist–hip ratio before and after the intervention (*P* > 0.05), as outlined in Table 3.

**Table 3:** Changes in pelvic floor muscle strength and waist-to-hip ratio before and after intervention

Group	Pelvic floor muscle strength		Waist–hip ratio	
	Before intervention	After intervention	Before intervention	After intervention
Intervention group (*n* = 53)	2.51 ± 0.50	2.64 ± 0.59	0.828 ± 0.024	0.804 ± 0.104
Control group (*n* = 53)	2.53 ± 0.54	2.71 ± 0.63	0.823 ± 0.026	0.817 ± 0.024
*T*-value	0.186	0.635	0.934	0.918
*P*-value	0.853	0.527	0.353	0.361

### 3.3 Comparison of DRA between the two groups

Throughout the study, no cases were lost, and all data were incorporated into the statistical analysis. Prior to the intervention, no notable difference in IRD was observed between the two groups of women (*P* > 0.05). Following the intervention, IRD measurements above, at, and below the umbilicus exhibited a significant reduction in both groups compared to pre-intervention values. Also, the intervention group displayed a notably greater reduction in IRD at all measured points compared to the control group, indicating statistical significance (*P* < 0.05), as depicted in Table 4.

**Table 4:** The diastasis recti abdominis distance in the two postpartum women groups (cm)

Group	Above the umbilicus 2 cm distance				Umbilicus level				Below the umbilicus 2 cm distance			
	Before intervention	After intervention	*P*	Cohen *d* (within groups)	Before intervention	After intervention	*P*	Cohen *d* (within groups)	Before intervention	After intervention	*P*	Cohen *d* (within groups)
Intervention group	3.74 ± 0.59	1.62 ± 0.55	<0.001	3.717	4.31 ± 0.50	2.28 ± 0.51	<0.001	4.020	3.39 ± 0.63	1.43 ± 0.54	<0.001	3.341
Control group	3.77 ± 0.61	3.56 ± 0.63	0.035	0.334	4.27 ± 0.62	3.98 ± 0.46	<0.001	0.531	3.50 ± 0.69	3.19 ± 0.60	<0.001	0.479
*P*-value	0.809	0.000			0.732	0.000			0.422	0.000		
Cohen *d* (between groups)		3.281				3.500				3.083		

Small to medium and very large positive, clinically meaningful effects were found for IRD at all measured points in the control group and the intervention group, respectively. The magnitude of the between-group effects was very large in size, favoring the intervention group, as depicted in Table 4.

## 4 Discussion

This study highlights the efficacy of core muscle strength stability-oriented breathing training in effectively enhancing postpartum diastasis recti. This intervention demonstrates applicability across various age groups and parity statuses among postpartum women, with no reported adverse reactions and high compliance rates. During pregnancy, the transversus abdominis muscle undergoes considerable stretching, while the rectus abdominis muscles experience fatigue, thereby accelerating the stretching and separation of the linea alba. The linea alba, located centrally between the rectus abdominis muscles on either side, plays a pivotal role in DRA formation [14]. This condition indirectly contributes to various postpartum issues such as stress urinary incontinence and pelvic organ prolapse [15]. Addressing DRA promotes the recovery of associated tissues and muscle groups in postpartum women, consequently enhancing their overall quality of life [16]. In clinical practice, the predominant treatments for DRA involve neuromuscular electrical stimulation and exercise therapy [17]. Neuromuscular electrical stimulation serves to activate and stimulate muscles, facilitating relaxation in neurologically intact muscles, and contributes to passive muscle strengthening, thereby enhancing muscle contraction and mobility [18,19].

DRA represents a manifestation of an underlying issue characterized by a decline in the core strength of the transversus abdominis muscle [20]. Consequently, addressing this condition necessitates a primary focus on strengthening the transversus abdominis muscle rather than solely targeting the rectus abdominis. Effective enhancement of the transversus abdominis muscle is essential for addressing and improving DRA [21]. Based on core breathing theories, diaphragmatic movements influence the contraction of the transversus abdominis muscle, thereby impacting the functional activity of the rectus abdominis [22]. Consequently, breathing training may hold the potential for improving symptoms associated with DRA in postpartum women [23]. Such training could prompt early activation of deep core muscle contractions, thereby stabilizing the trunk in anticipation of functional activities in postpartum women with DRA [24]. It is worth noting that abdominal curl exercises should be avoided in cases of DRA, as they have the potential to exacerbate the separation [25].

Exercise training, as a prevalent rehabilitation approach for patients with DRA, primarily emphasizes abdominal and lower limb aerobic exercises [26]. This regimen proves beneficial in addressing various postpartum issues, including urinary incontinence, obesity, DRA, constipation, and lower back pain [27]. However, for patients experiencing DRA with pelvic floor muscle strength <3, rendering them temporarily unable to engage in active DRA training, passive strengthening of the rectus abdominis muscle via abdominal neuromuscular electrical stimulation may initially contribute to enhancing or maintaining the extent of DRA [28]. This approach can be supplemented with Kegel exercises to enhance pelvic floor muscle strength, thereby laying the groundwork for the subsequent initiation of active core muscle group training [29].

Early self-directed exercise training has demonstrated favorable outcomes in the treatment of postpartum diastasis recti [30]. However, such approaches require high patient compliance, as improper force application or breath holding during training sessions can worsen the severity of DRA. Prior studies have successfully implemented breathing training during the postpartum period, demonstrating its efficacy in enhancing pelvic floor muscle function recovery and addressing stress urinary incontinence. The present study further validates breathing training as the most optimal method for enhancing transversus abdominis muscle strength in cases of postpartum DRA. It can serve as a supplementary training modality for postpartum women affected by DRA, thereby contributing to the rehabilitation of this condition.

Previous studies have indicated that various interventions, such as pelvic floor neuromuscular electrical stimulation combined with rehabilitation massage, neuromuscular electrical stimulation alongside Kegel exercises, and pelvic floor neuromuscular electrical stimulation in conjunction with suspension training, have the potential to ameliorate postpartum diastasis recti [31,32,33]. However, the breathing training approach introduced in this study boasts versatility, simplicity in learning, convenience, and safety, rendering it more favorable for widespread adoption among postpartum women.

The study is constrained by limitations due to its inability to accurately assess the intensity of breathing training. Through the incorporation of wearable devices or alternative methodologies to precisely measure the intensity of the breathing exercises, enhanced accuracy and reliability of the findings could be attained.

## 5 Conclusion

Core muscle strength stability-oriented breathing training proves effective in reducing IRD in postpartum women, thereby facilitating the recuperation of associated muscle groups and enhancing overall physical condition.

## Abbreviations

IRD: inter-recti distance
DRA: diastasis recti abdominis
